# Drug allergy evaluation in children with suspected mild antibiotic allergy

**DOI:** 10.3389/falgy.2022.1050048

**Published:** 2022-12-05

**Authors:** Nikolaos Kitsos, Dimitrios Cassimos, Grigorios Trypsianis, Ioannis Xinias, Emmanouil Roilides, Ioanna Grivea, Elpis Mantadakis, Antigoni Mavroudi

**Affiliations:** ^1^ 3rd Pediatric Clinic, Aristotle University of Thessaloniki, Thessaloniki, Greece; ^2^ Department of Medicine, Democritus University of Thrace, Alexandroupolis, Greece; ^3^ School of Health Sciences, University of Thessaly, Larisa, Greece

**Keywords:** drug allergy, skin tests, drug provocation test, children, graded challenge

## Abstract

**Background:**

Adverse antibiotic reactions caused by an immunological mechanism are known as allergic reactions. The percentage of reported antibiotic allergies is likely to differ from the one validated after a drug provocation test (DPT) with the culprit antibiotic. This study aimed to compare the percentage of children who were thought to be allergic to a certain antibiotic with those who have a true allergy, as confirmed by DPTs. We also validated Skin Prick Tests (SPTs) and Intradermal Tests (IDTs) by assessing their sensitivity and specificity, in diagnosing antibiotic allergies using DPT as the gold standard. Furthermore, we investigated epidemiological risk factors such as personal and family history of atopic disease and eosinophilia.

**Methods:**

Children with a history of possible allergic reaction to an antibiotic underwent a diagnostic procedure that included: (1) Eosinophil blood count, (2) SPTs, (3) IDTs and (4) DPTs. The parameters were compared with Pearson's Chi-Square and Fisher's Exact Test. Several risk factors that were found significant in univariate analysis, such as personal and family history of atopic disease, and positive SPTs and IDTs were examined with multiple logistic regression analysis to see if they were related to a higher risk for a positive DPT.

**Results:**

Semi-synthetic penicillin was the most common group of antibiotics thought to cause allergic reactions in this study. Overall, 123 children with a personal history of an adverse reaction to a certain antibiotic, were evaluated. In 87.8% of the cases, the symptoms had occurred several hours after administration of the culprit antibiotic. Both SPTs and IDTs had low sensitivity but high specificity. Moreover, they had a high positive predictive value (PPV). In contrast, eosinophilia was not recognized as a risk factor. Seventeen patients (13.8%) had a true antibiotic allergy, as confirmed by a positive DPT. A positive IDT was a strong predictor of a positive DPT, along with a positive personal and family history of atopy.

**Conclusion:**

SPTs and IDTs are very reliable in confirming antibiotic allergy when found positive. A negative result of a SPT highly predicts a negative DPT. A positive IDT and a positive personal and family history of atopy were recognized as significant risk factors for antibiotic allergy.

## Introduction

Beta-lactam antibiotics are the most common cause of drug-induced hypersensitivity reactions in children. However, non-beta-lactam (NBL) antibiotics can also cause hypersensitivity responses, estimated to be between 1% and 3% of the general population (1). Most articles on antibiotic allergy have focused on beta-lactam hypersensitivity, whereas reactions to NBLs have been presented mainly as case reports (2). The management of antibiotic allergy begins with identifying the culprit antibiotic based on a comprehensive medical history. A detailed medical history for allergies and a meticulous physical examination are critical for accurately diagnosing drug-induced reactions (3).

To identify immediate allergic reactions to antibiotics, SPTs and IDTs are available. IDTs are used for immediate as well as late reactions and are evaluated immediately and after 24 and 72 h. For late reactions, an infiltrating erythematous wheal is characterized as a positive reaction. Immediate positive reactions consist of a wheal with surrounding erythema. However, only the wheal is measured. A SPT is considered positive if the wheal has a minimum diameter of 3 mm (4, 5). However, these are mainly standardized for beta-lactam antibiotics and not for other antibiotic groups. Skin and/or provocation tests may be used to confirm reactions (6–9).

Even though the percentage of children with reported allergies vs. those confirmed by DPTs has been determined previously, in some populations, the validity of SPTs and IDTs has not been adequately defined in the current literature. Therefore, this study aimed to provide additional evidence regarding the diagnostic value of SPTs and IDTs in diagnosing antibiotic allergies. Furthermore, several risk factors, such as peripheral eosinophilia and personal and family history of atopic disease were investigated for an association with the risk of true antibiotic allergy.

## Materials and methods

**Figure 1:**
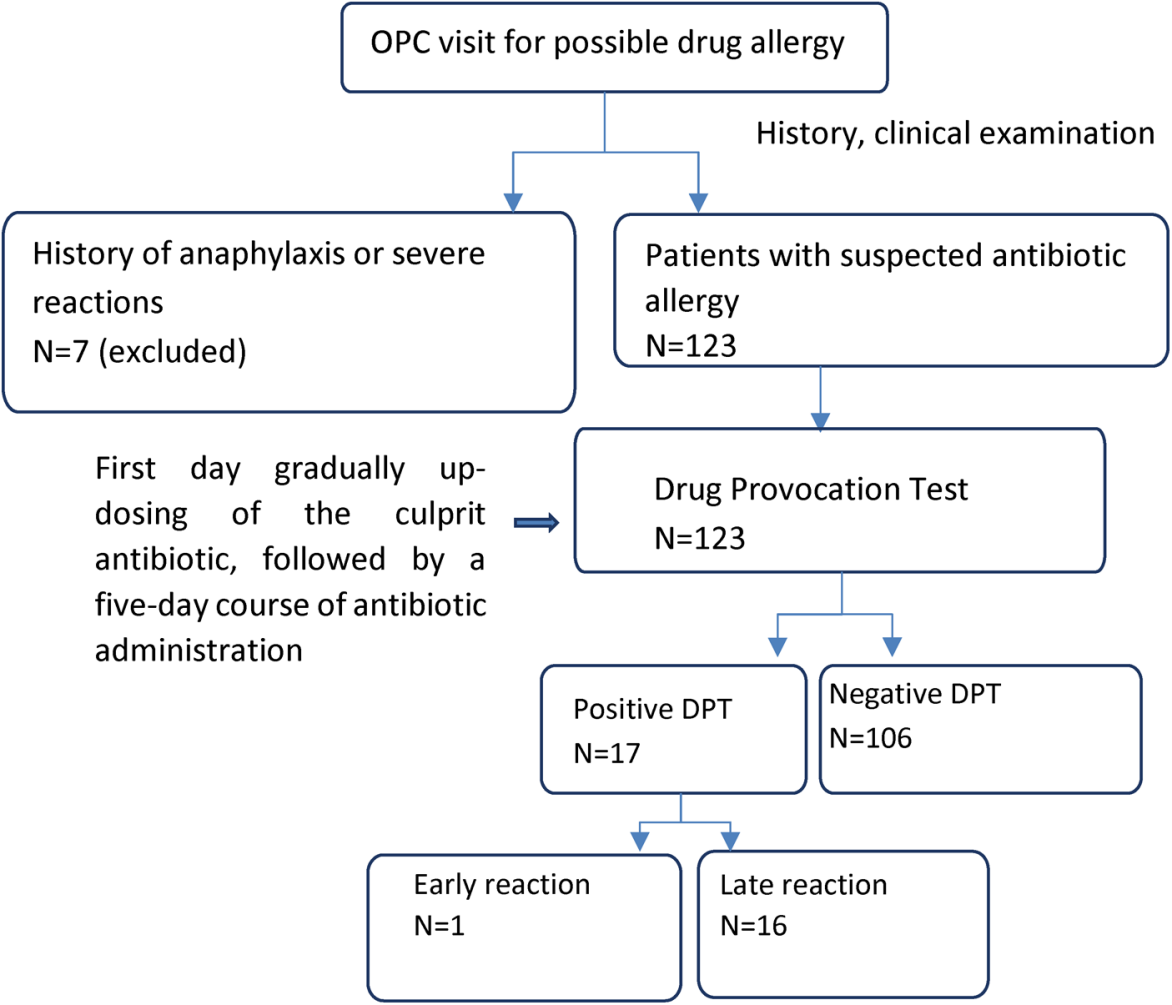
Protocol illustrating the procedure of the drug provocation test.

Our study took place at the University Pediatric Allergy Department of the Hippokrateion Hospital of Thessaloniki. We recruited children aged 1 to 15 years old, who were referred with a clinical diagnosis of antibiotic allergy (Figure 1). The bioethics committee of the Aristotle University of Thessaloniki approved the research design and protocol (number of approval 424, 20/01/22). The initial selection of the children was carried out using the standardized European Academy of Allergy and Clinical Immunology (EAACI) questionnaire of the European Network of Drug Allergy (ENDA) (10). The questionnaire included a detailed medical and allergy history (both personal and family history), the initial symptoms of the allergic reaction, the culprit antibiotic, the cause of antibiotic treatment, the treatment of the hypersensitivity reaction, the organ systems affected by the reaction, and finally the route and dose of the administered drug.

### Classification of reactions

Drug hypersensitivity reactions that occurred 1–6 h after the drug administration were considered immediate reactions, whereas those that occurred after 6 h or more were considered late reactions.

### Exclusion criteria

The exclusion criteria were: (1) Age: infants < 1-year-old and teenagers > 15-years-old, (2) Previous anaphylaxis to the culprit antibiotic, (3) Severe skin and mucosal reactions (e.g., drug reaction with eosinophilia and systemic symptoms, Stevens-Johnson syndrome, toxic epidermal necrolysis, acute generalized exanthematous pustulosis) to the culprit antibiotic (Figure 1).

### Skin tests

We performed SPTs and IDTs on all subjects. Both SPTs, IDTs, and DPTs were performed by medical and nursing personnel trained to identify and treat anaphylactic reactions. The concentration of the drugs used for the skin tests had been proven to be nonirritating in previous studies, shown in Table 1 (11, 12).

**Table 1:** Nonirritating test concentrations for antibiotics.

Antibiotic	SPT concentration	IDT concentration
Amoxicillin	20 mg/ml	20 mg/ml
Amoxicillin/clavoulanic acid	20 mg/ml/4 mg/ml	20 mg/ml/4 mg/ml
Cephalosporins	2 mg/ml	2 mg/ml
Clarithromycin	50 mg/ml	0.5 mg/ml
Azithromycin	100 mg/ml	0.1 mg/ml

SPT, skin prick test; IDT, intradermal test.

### Drug provocation tests (DPTs)

We performed DPTs on all subjects. The initial dose of the examined antibiotic administered to the subjects was 1/4 of the maximum single unit dose, followed within 30 min intervals by administration of half of the maximum single unit dose and finally, the maximum single unit dose. The latter was calculated according to the patient's age and weight. The culprit antibiotic was subsequently administered at home for four more consecutive days (13).

### Statistical analysis

Statistical analysis was performed using IBM Statistical Package for the Social Sciences (SPSS), version 19.0 (IBM Corp., Armonk, NY, United States). Quantitative variables are expressed as mean ± standard deviation (SD) and qualitative variables are expressed as absolute and relative (%) frequencies. The association between qualitative variables was assessed using the chi-square test and Fisher's exact test. For the evaluation of the independent effect of children's characteristics on the risk of being diagnosed with an antibiotic allergy, multiple stepwise logistic regression analyses were used and adjusted for all children's characteristics of interest, i.e., sex, age, history of atopic disease, eosinophilia, positive SPT, and IDT. Odds ratios (OR) with their 95% confidence intervals (CI) were estimated as the measure of the above associations. All tests were two-tailed and statistical significance was set at *p* values <0.05.

## Results

### Demographic characteristics

Nine hundred and ninety-one (991) children visited the University Pediatric Allergy Department between December 2017 and February 2020. Overall, 123 children reported hypersensitivity reactions to antibiotics, 73 boys and 50 girls. The mean age was 7.5 years (SD ± 4.5). Thirty-nine children (31.7%) had an atopic background (eczema, food allergy, allergic rhinitis or allergic asthma). Demographics of the study population are shown in Table 2. The most common diagnosis was a respiratory tract infection (95.1%).

**Table 2:** Demographics of study population.

	Total sample	Positive DPT	*p* value	OR (95% CI)
No of children	123	17 (13.8)		
Sex			0.122	
Female	50 (40.7)	4 (8.0)		Ref.
Male	73 (59.3)	13 (17.8)		2.49 (0.76–8.15)
Age			0.400	
≤6 years	55 (44.7)	6 (10.9)		Ref.
>6 years	68 (55.3)	11 (16.2)		1.58 (0.54–4.57)
Personal history of atopic disease			0.366	
No	84 (68.3)	10 (11.9)		Ref.
Yes	39 (31.7)	7 (17.9)		1.62 (0.57–4.63)
Family history of atopic disease			0.027	
No	86 (69.9)	8 (9.3)		Ref.
Yes	37 (30.1)	9 (24.3)		3.13 (1.10–8.92)
History of atopic disease			0.013	
No	62 (50.4)	7 (11.3)		Ref.
Personal only	24 (19.5)	1 (4.2)		0.34 (0.40–2.94)
Family only	22 (17.9)	3 (13.6)		1.24 (0.29–5.29)
Personal and family	15 (12.2)	6 (40.0)		5.24 (1.43–19.19)
Eosinophilia			0.683	
No	118 (95.9)	16 (13.6)		Ref.
Yes	5 (4.1)	1 (20.0)		1.59 (0.17–15.18)
Skin prick test			<0.001	
Negative	121 (98.4)	15 (12.4)		–
Positive	2 (1.6)	2 (100.0)		–
Positive intradermal test			<0.001	
Negative	118 (95.9)	13 (11.0)		Ref.
Positive	5 (4.1)	4 (80.0)		32.31 (3.35–311.41)

### Culprit antibiotic

The analysis showed that most reported reactions were due to the penicillin group (58.6%). In 30.1% (*n* = 37) of the cases, the suspected drug was amoxicillin, while in 28.5% (*n* = 35) of the cases, the suspected drug was the combination of amoxicillin with clavulanic acid. Cephalosporines and macrolides represented 27.6% (*n* = 34) and 13.8% (*n* = 17) of suspected antibiotic allergy, respectively.

### Type of reaction

In our research, 87.8% (*n* = 108) of the patients had symptoms that occurred several hours after the antibiotic administration, and in 12.2% (*n* = 15) of the cases, symptoms appeared in the first hour after the antibiotic administration. We also found that most children (92.7%, *n* = 114) had symptoms from the skin, such as urticaria, angioedema, flushing, or maculopapular rash. A percentage of 6.5% had symptoms from the gastrointestinal tract, such as vomiting, nausea, and abdominal pain.

### Outcome of DPT

Seventeen out of 123 patients (13.8%) had a positive DPT. One out of seventeen patients (5.8%) with a positive DPT developed a severe systemic reaction that fulfilled the diagnostic criteria of anaphylaxis. This single patient was a 12-year-old girl with a suspected clarithromycin allergy. She developed a fine papular exanthema 2 h after the intake of the third dose of clarithromycin. During DPT, 30 min after the intake of the last dose, she developed a maculopapular rash over large areas of her body, along with abdominal pain, severe vomiting, diarrhea, and malaise. She received symptomatic therapy with administration of adrenaline, antihistamines, and intravenous fluids. Most of the patients, i.e., 94.2% of them (16 out of 17), presented a delayed positive DPT with mild self-limited symptoms. Fifteen out of 16 patients presented the symptoms at home during the second day of the DPT (Supplementary Table S1). These patients had symptoms from the skin, corresponding to a grade 1 systemic allergic reaction according to the World Allergy Organization grading system (14).

### Skin prick tests and intradermal tests

It was then investigated whether the SPTs and the IDTs are valid for allergy diagnosis. For this purpose, we calculated sensitivity, specificity, PPV, and negative predictive value (NPV) compared to the DPTs outcome, which is considered the “gold standard” for the diagnosis of drug allergy for all antibiotic groups, as well as for beta-lactams and macrolides, respectively. SPTs were found to have a sensitivity of 11.8% (2/17 = 0.118), a specificity of 100% (106/106 = 1), a PPV of 100% (2/2 = 1), a NPV of 87.6% (106/121 = 0.876) and accuracy 87.8% (80.7–93.0), respectively. IDTs had a sensitivity of 23.5% (4/17 = 0.235), a specificity of 99.1% (105/106 = 0.991), a PPV of 80% (4/5 = 0.8), a NPV of 89% (105/118 = 0.890), and accuracy of 88.6% (81.6–93.6), respectively. These values (sensitivity, specificity, PPV, and NPV) were also calculated for penicillins, cephalosporins, and macrolides separately, as shown in Table 3.

**Table 3:** Sensitivity, specificity and predictive values of penicillins, cephalosporins and macrolides.

	Penicillins		Cephalosporines		Macrolides	
	SPT	IDT	SPT	IDT	SPT	IDT
Sensitivity (%)	0.0 (0.0–36.9)	0.0 (0.0–36.9)	28.6 (3.7–80.0)	57.1 (18.4–90.1)	0.0 (0.0–84.2)	0.0 (0.0–84.2)
Specificity (%)	100.0 (94.4 –100.0)	100.0 (94.4–100.0)	96.3 (81.0–99.9)	96.3 (81.0–99.9)	100.0 (78.2–100.0)	100.0 (78.2–100.0)
PPV (%)	–	–	66.7 (17.4–95.0)	80.0 (34.5–96.8)	–	–
NPV (%)	88.9	88.9	83.9 (76.4–89.3)	89.7 (78.6–95.3)	88.2	88.2
Accuracy (%)	88.9 (79.3–95.1)	88.9 (79.3–95.1)	82.4 (65.5–93.2)	88.2 (72.6–96.7)	88.2 (63.6–98.5)	88.2 (63.6–98.5)

PPV, positive predictive value; NPV, negative predictive value.

### Risk factors for antibiotic allergy

Univariate statistical analysis revealed that a positive DPT was more frequent in children with a family history of atopic disease (*p *= 0.027) and even more in children with a co-existing personal history of atopic disease (*p *= 0.013).

A correlation was also observed between positive SPTs and positive DPTs (*p *< 0.0001), and positive IDTs and positive DPTs (*p *< 0.0001).

A tendency towards a high frequency of positive DPTs was observed in male subjects (*p *= 0.125).

No statistically significant correlation was found between eosinophilia and positive DPTs (Table 1).

Multiple logistic regression analysis showed that the simultaneous presence of a personal and family history of atopy (*p *= 0.002) and positive intradermal tests (*p *= 0.002) were the two independent predictors for a positive challenge test in children with possible drug allergies. Specifically, children with positive intradermal tests were 66 times more likely to have a positive challenge (Adjusted Odds Ratio – aOR: 66.14, 95% CI: 4.56–960.18), and children with a personal and family history of atopy were 11 times more likely to have a positive challenge (aOR: 11.21, 95% CI: 2.45–51.27).

## Discussion

In this study, 123 children with a clinical diagnosis of antibiotic allergy were evaluated for true drug allergy by a process that included history, physical examination, skin tests (SPTs and IDTs), and finally, DPTs. True antibiotic allergy was confirmed in 13.8% of the cases. In most cases, a semi-synthetic penicillin was the culprit antibiotic, followed by cephalosporines and macrolides.

Self-reported drug allergy has a prevalence of 8% in the general population (15). Beta-lactam allergy has been estimated to occur in up to 15% of hospitalized patients (16). While drug allergy is relatively uncommon, many children are labeled as “allergic” to various medications, particularly antibiotics. This study evaluated a group of 123 children with reported antibiotic allergy for true antibiotic allergy, which was confirmed in 17 patients (13.8%). Therefore, false antibiotic allergy had a high reported prevalence of more than 86%. Antibiotic allergy overdiagnosis is a significant healthcare issue. Thus, due to the overuse of the term “allergy”, e.g., in the presence of skin rashes most commonly caused by viral infections, many children are falsely labeled as allergic to an antibiotic. This can lead to the extensive use of alternative antibiotics (17). Many children who carry the label of being allergic to an antibiotic are treated with alternatives that may be less effective, leading to difficult to treat infections and contributing to the development of bacterial resistance (15, 18, 19).

SPTs and IDTs demonstrated relatively low sensitivity but high specificity. Positive skin tests, both SPTs and IDTs, strongly predicted a positive DPT to the culprit antibiotic. A positive personal and family history of allergy was recognized as a risk factor for true antibiotic allergy.

Delayed-type hypersensitivity reactions occurred in 94.2% of the patients with a positive DPT. Only one child (0.8%) developed true anaphylaxis out of the 123 patients. This finding is consistent with previous studies showing a low probability of potentially life-threatening reactions during DPT (20). Of interest, this female patient had no history of a severe reaction during her previous exposure to the antibiotic that had been implicated. Therefore, patients who report mild symptomatology after taking an antibiotic to which they experienced an allergic reaction may have more severe systemic symptomatology when a diagnostic DPT is performed.

We sought to assess the accuracy of SPTs and IDTs, in predicting antibiotic allergy and, in comparison to DPT, which is considered the “gold standard” for a definite diagnosis. Sensitivity was 11.8% and 23.5% for SPTs and IDTs, respectively, while specificity was 100% and 99.1%, respectively. Moreover, the PPV and NPV were high for SPTs and IDTs (PPV: 100% and 80%, and NPV 87.6% and 89% for SPTs and IDTs, respectively). Similarly, a systematic review and a prospective study assessing the diagnostic accuracy of SPTs in penicillin-allergic patients reported a high specificity but a low sensitivity of less than 50% in predicting hypersensitivity reactions. Overall, the results of that review suggested that, at least in patients reporting a delayed mild reaction, skin tests may have a high specificity (97.4%) and NPV, but a low PPV and sensitivity (19.3%) in identifying patients who will develop a hypersensitivity reaction when exposed to penicillin (21, 22).

Another study by Yoon et al. reported that the IDT for cephalosporins had a sensitivity of 0%, a specificity of 97.5%, a NPV of 99.7%, and a PPV of 0% when challenged with the same drugs that were positive in the skin test (23). The present study showed that IDTs had high specificity in predicting a positive DPT.

Our study also revealed that the simultaneous presence of personal and family history of atopic disease and a positive IDT were the two independent factors related to a positive DPT, and consequently true drug allergy. As reported in 1993 by Gadde J et al., previous findings showed no association between penicillin allergy and atopy (24), although patients with asthma were more prone to severe reactions (25). However, there are several studies suggesting that drugs causing delayed hypersensitivity adverse reactions in the presence of HLA alleles include allopurinol, antiretrovirals (namely abacavir and nevirapine), aromatic amine anticonvulsants (in particular carbamazepine and phenytoin), and sulfonamides (25–27). An immunological response to certain drug antigens may be triggered in patients carrying specific HLA alleles, leading to T-cell activation and clonal expansion (28). Moreover, recent studies show an association between the HLA-DRB1∗10:01 allele and hypersensitivity reactions to penicillin (29).

Α limitation of the current study is that the statistical analysis concerning the validity of the tests, SPTs and IDTs, specifically the sensitivity, specificity, PPV, and NPV were performed on a relatively small patient sample, in whom the diagnosis of antibiotic allergy was made after the positive challenge test, and therefore their statistical power is low. Re-evaluation in larger patient samples is required. In addition, the inclusion of these patients in future meta-analyses will lead to more reliable results.

In summary, in the current study, true antibiotic allergy was confirmed in a small number of patients, 13.8% of those with a clinical diagnosis of antibiotic allergy. Semisynthetic penicillins were the most frequently implicated antibiotics, followed by cephalosporins and macrolides. Most allergic reactions to antibiotics are of delayed type, with the skin being the most frequently affected organ, followed by the gastrointestinal tract. SPTs and IDTs had high specificity and positive and negative predictive values. A positive IDT was a strong predictor of a positive DPT. Regarding IDTs, there is no published data showing a statistical association between skin tests and DPTs outcomes. Interestingly enough, the simultaneous presence of a positive personal and family history of atopy was also associated with an increased risk for a positive DPT, and subsequently the diagnosis of true antibiotic allergy.

## Data Availability

The original contributions presented in the study are included in the article/Supplementary Material, further inquiries can be directed to the corresponding author/s.
