# Supplementary material for: Drug allergy evaluation in children with suspected mild antibiotic allergy
Source: Front Allergy. 2022 Dec 5;3:1050048. doi: 10.3389/falgy.2022.1050048 (PMC9784922; doi:10.3389/falgy.2022.1050048)
Supplement: Supplementary file 1 [file Table1.pdf]

Supplementary Material

Supplementary Table S1. Skin tests, chronology and clinical presentations of patients with confirmed antibiotic allergy after DPT

Patients	SPT	IDT	DPT		
			Timing		Symptoms
			Immediate (1-6 hours from drug administration)	Delayed (>6h from drug administration)	
1	negative	positive		positive	Maculopapular rash
2	negative	negative		positive	Maculopapular rash
3	negative	negative		positive	Maculopapular rash
4	negative	negative		positive	Maculopapular rash
5	negative	negative		positive	Maculopapular rash
6	negative	negative		positive	Maculopapular rash
7	negative	negative		positive	Maculopapular rash
8	negative	negative		positive	Maculopapular rash
9	positive	positive		positive	Maculopapular rash
10	negative	negative		positive	Urticaria
11	negative	negative		positive	Maculopapular rash
12	negative	negative		positive	Maculopapular rash
13	positive	positive		positive	Urticaria
14	negative	negative		positive	Maculopapular rash
15	negative	positive		positive	Urticaria
16	negative	negative		positive	Maculopapular rash
17	negative	negative	positive		Anaphylaxis
